# Personalized Diagnoses for Those Born with Congenitally Malformed Hearts

**DOI:** 10.3390/jpm15030102

**Published:** 2025-03-04

**Authors:** Adrian C. Crucean, Diane E. Spicer, Justin T. Tretter, Rohit Loomba, Robert H. Anderson

**Affiliations:** 1 Department of Paediatric Cardiac Surgery, Birmingham Women’s and Children’s Hospital, Birmingham B4 6NH, UK; 2 Heart Institute, Johns Hopkins All Children’s Hospital, St Petersburg, FL 33701, USA; spicerpath@hotmail.com; 3 Department of Pediatric Cardiology, Cleveland Clinic Children’s, and The Heart, Vascular, and Thoracic Institute, Cleveland Clinic, Cleveland, OH 44195, USA; trettej3@ccf.org; 4 Division of Pediatric Cardiology, Lurie Children’s Hospital, Chicago, IL 60611, USA; rohit.loomba@gmail.com; 5 Biosciences Institute, Newcastle University, Newcastle-upon-Tyne NE2 4HH, UK; sejjran@ucl.ac.uk

**Keywords:** visceral heterotaxy, interatrial shunting, deficient ventricular septation, bicuspid aortic valve, unicuspid and unicommissural aortic valve, nomenclature

## Abstract

**Background/Objectives**: It is increasingly realized that the advances in diagnosis and treatment for those born with congenitally malformed hearts have now resulted in avoidance of morbidity being equally as important as avoiding postoperative mortality. Detailed personalized diagnoses will now be key to achieve such improvements. **Methods**: We have reviewed our own experience in diagnosing major phenotypic variations on selected congenital cardiac malformations, showing that the ability to personalize the findings is at hand, although not always to date universally employed. **Results**: We have chosen four categories to illustrate how the definitions now provided by the International Nomenclature Society, and incorporated in the 11th iteration of the International Classification of Disease, make it possible to provide personalized diagnoses. The lesions chosen for review are the arrangement of the atrial appendages, the lesions permitting interatrial shunting, the options in the setting of deficient ventricular septation, and the abnormal morphology of the aortic root. We show that not all centers, as yet, are taking advances of these opportunities at hand to tailor the chosen treatments. **Conclusions**: Detailed phenotypic definitions have now been provided for all the major congenital cardiac malformations. Use of these definitions should now provide personalized medicine for all those born with malformed hearts. As yet, the definitions are not used to their full effect.

## 1. Introduction

It is increasingly recognized that not all patients, even when they have the same phenotypic lesion, are necessarily the same. In many fields, the personalized approach required to provide the optimal treatment is dependent on analysis of the genetic background of the patient. This is true for those born with congenitally malformed hearts, but much simpler problems currently prevent the optimal implementation of the personalized approach to these patients. This is because, despite the production of detailed definitions of the various phenotypes existing within a given known congenital cardiac malformation, as yet not all centers, even those with World-class reputations, are employing these definitions to their full extent. Thus, despite the detail offered in the definitions, now enshrined within the 11th iteration of the International Classification of Disease produced by the World Health Organisation [1], disagreements continue with regard to the words used to account for the phenotypic variations. It may seem that the use of different words to describe the same entity is of little importance if those working in a given center are themselves aware of the meaning of the word in their center. This is not necessarily the case. For example, one World-class center is currently promoting the use of septation for children born with double inlet ventricle [2]. The surgical results are outstanding. As yet, however, the team have not obtained the reduction in morbidity they had hoped to achieve. It can be argued that this is because it is not always possible to correlate the words used in their center to describe the lesions being treated with those now used in other World-class centers. This can make it difficult for those used to different categorisations and nomenclatures to take full advantage of their findings. This potential problem is not limited to the diagnosis of patients born with functionally univentricular hearts, although it is widespread still to encounter accounts of such patients as having “single ventricles”, when the images now available show that almost all such individuals have one big and one small ventricle. Another example is found in their approach to patients having straddling and overriding of the tricuspid valve. This lesion is still, seemingly, described in their center as an “atrioventricular canal defect”, despite the fact that such patients have separate right and left atrioventricular junctions. In this review, in which we focus on four specific groups of lesions, we show how the appropriate use of words might become an integral part of the advancement of personalized medicine.

## 2. Materials and Methods

The eleventh iteration of the International Classification of Disease, prepared on behalf of the World Health Organisation, now contains over 300 definitions providing details of the phenotypic variability to be found amongst children born with congenitally malformed hearts [1]. These definitions were produced after long and detailed discussions between members of the International Society for Nomenclature for Pediatric and Congenital Heart Disease [3]. In addition to the definitions produced, because of the use of different systems of categorization in different institutes, it also proved necessary to provide lists of synonyms for many of the words used. These synonyms, however, are not always suitable for providing the details now required to optimize personalized diagnoses. Nor are all the words, despite the best efforts of the Society, used in logical or linguistically optimal fashion. In this review, we take advantage of our own experience derived from examination of archives of congenitally malformed hearts to illustrate the problems that still exist when words are not used in their most appropriate fashion to account for the phenotypic variations. The lesions we have chosen for discussions are the arrangements of the atrial appendages; the channels that permit shunting, on the one hand, between the atrial chambers, and on the other hand between the ventricles; and the malformations which involve the leaflets of the aortic root.

## 3. Results

### 3.1. Arrangement of the Atrial Appendages

**Figure 1:**
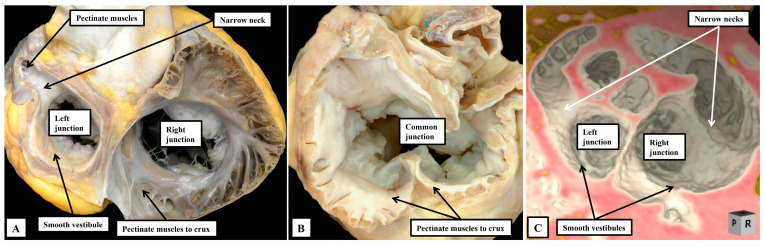
(**A**,**B**) have been prepared by cutting back the atrial myocardium so as to permit assessment of the atrioventricular junctions from above, with the left side seen to the right hand of the observer. (**C**) is a virtual dissection of a computed tomographic dataset producing a comparable view of the atrioventricular junctions. (**A**) shows the usual arrangement, with (**B**,**C**) showing the arrangements as seen when the atrial appendages are isomeric, with (**B**) showing right isomerism, and (**C**) left isomerism.

It is now well recognised that the starting point for personalized diagnosis in patients with congenitally malformed hearts is the establishment of the arrangement of the atrial chambers [4]. This has not always been easy in the clinical setting. Describing the variants has also been far from uniform. There is still the tendency to describe the arrangements using Latin words such as “situs solitus”. This, of course, means no more than usual arrangement. The unfortunate corollary of the use of Latin is that the mirror-imaged variant is still most frequently described as being “situs inversus”. This is confusing, since the bodily contents are not themselves upside-down. Patients with mirror-imaged bodily arrangement, however, are exceedingly rare. It is much more frequent to find individuals in which the atrial chambers, and the bodily organs, are found neither in their usual arrangement, nor their mirror-imaged variant. Appropriate description of these individuals, who usually harbour the most severe combinations of intracardiac lesions [5], has long been problematic. For quite some time, the patients were described as having “splenic syndromes” [6]. When the concept of segmental analysis first emerged, they were considered to have an ambiguous arrangement [7]. When the arrangement of the atrial chambers is judged according to the extent of the pectinate muscles within the appendages relative to the atrial vestibules, however, there is not ambiguity with regard to the different patterns. In the normal arrangement, the pectinate muscles in the morphologically right atrium encircle the smooth-walled vestibule, extending to the cardiac crux. In the morphologically left atrium, in contrast, the pectinate muscles are confined within the tubular appendage, leaving a smooth inferior vestibule (Figure 1A). In the mirror-imaged variant, the vestibule encircled by the pectinate muscles is found on the left side. In the arrangements previously interpreted as creating problems, the extent of the pectinate muscles shows that the atrial appendages are isomeric, showing the features of either right isomerism (Figure 1B), or left isomerism (Figure 1C).

From the stance of analysis of the heart itself, therefore, assessment of the morphology of the appendages, when based on the extent of the pectinate muscles relative to the atrial vestibules (Figure 1), permits all individuals to be distinguished as having either the usual arrangement, its mirror-image, or one of the two isomeric arrangements [4].

The problem for those clinicians seeking personalized medicine, of course, is that the features as shown in the autopsy room (Figure 1) are not always manifested as obviously during life. It can be difficult using echocardiography, still the most-used clinical diagnostic tool, to demonstrate the extent of the pectinate muscles. In contrast, cardiac computed tomography, when obtained, often readily reveals this detail (Figure 1C). There are additional clues, nonetheless, that should help in identifying the variants during life. For example, the coronary sinus is always present within the morphologically left atrioventricular junction. Absence of this feature on both sides, therefore can serve to identify right isomerism, if this is suspected. This feature, however, does not help in distinguishing left isomerism. There may be features suggestive of a coronary sinus within both junctions in the setting of left isomerism, but it is not possible to find fully-formed isomeric coronary sinuses. It remains a fact, therefore, that those clinicians seeking to individualise the atrial features must still depend on surrogates. Of these, establishment of splenic morphology was the initial approach to the arrangement still frequently described as “visceral heterotaxy” [8]. The abdominal organs, in fact, are truly “heterotaxic” in the setting of bodily isomerism. As has been pointed out by the group working in Las Vegas, Ref. [9] “heterotaxy” is any departure from the anticipated arrangement. These investigators emphasise, rightly, that those individuals with the mirror-imaged arrangement should also be considered to be “heterotaxic”, an approach which we fully endorse. It is only in the thoracic organs, specifically the lungs, the bronchuses, and as we have now shown, the atrial appendages, that we find the evidence for bodily isomerism. Bronchial morphology, furthermore, is a much better guide than the arrangement of the spleen to the likely presence of isomerism within the heart. The value of determining bronchial morphology for this purpose was shown long since by Landing and his colleagues [10]. We have also endorsed the value of the use of bronchial morphology in this setting [11]. We find it surprising, therefore, that Evans and his colleagues should consider ourselves to be outwith their newly established “canon” for the starting point of the personalized approach to the diagnosis of patients with congenitally malformed heart [9,12]. We are also in agreement with the group working in Las Vegas when they state that identification of the arrangement of the abdominal great vessels at the level of the diaphragm provides an excellent means of establishing bodily arrangement, Ref. [12] and is therefore a suitable indicator of likely atrial arrangement [13]. As good as are the findings as a marker for atrial arrangement, however, they are no more than that. And, as with the other surrogates, there is no certainty that atrial arrangement will be harmonious with the arrangement of the abdominal great vessels at the level of the diaphragm. Indeed, it is well established that interruption of the inferior caval vein, with left bronchial isomerism and multiple spleens, is frequently found in individuals having biliary atresia, but with usual arrangements in the heart [14]. In the final analysis, therefore, it is the arrangement of the atrial appendages themselves that should serve as the starting point of personalized cardiac diagnosis [4]. Only rarely will there be instances when disharmony is found between the arrangement of the atrial appendages and the remainder of the thoracic organs, with the location of the abdominal great vessels at the level of the diaphragm. In these rare cases, use of computed tomographic analysis will readily reveal the extent of the pectinate muscles relative to the atrial vestibules [15]. The focus on the atrial appendages then becomes the more important when it is remembered that the sinus node is a significant part of the morphologically right atrial appendage. This is why the node is right-sided when there is usual atrial arrangement, and left-sided in the setting of mirror-imagery. Of equal importance is that the sinus node is itself isomeric when there is right isomerism, Ref. [16] and grossly hypoplastic or absent when there is left isomerism [17]. And proper recognition of atrial arrangement has also been shown to be important in determining the arrangement of the atrioventricular conduction axis [18]. All of this points to the significance of establishing atrial arrangement as the starting point for sequential segmental analysis, and the beginning of the personalized approach.

### 3.2. The Channels Permitting Shunting Between the Atrial Chambers

**Figure 2:**
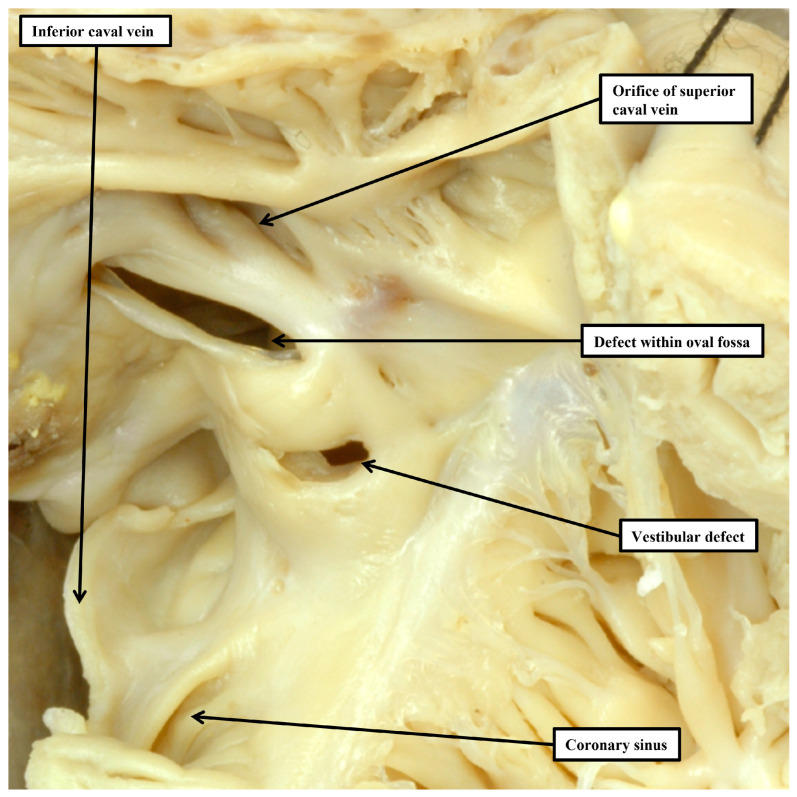
The right atrium in this heart has been opened to show the presence of the two channels permitting interatrial shunting that are true “septal defects”. There is a large defect in the floor of the oval fossa, present because the flap valve derived from the primary atrial septum, is insufficiently large to overlap the rims of the fossa. In addition, there is a second vestibular defect in the antero-inferior buttress, which is the true second atrial septum (see text for further discussion).

Once having established the arrangement of the atrial appendages, the next steps of the personalized approach are to determine the manner of the connections between the atrial and ventricular chambers, and similarly between the ventricles and the arterial trunks [4]. In this current review, we have neither the time nor the space to discuss the controversies that do still continue regarding how best to account for these features. Instead, we will discuss the problems that still exist in accounting for the channels that permit shunting between the chambers at atrial and ventricular levels. We begin with a discussion of the options for interatrial shunting. At first sight, this might seem very straight forward, since it might be intuitive that all such channels are “atrial septal defects”. Indeed, in many textbooks, particularly those written for practitioners in adult medicine, the various lesions are still described on this basis. In fact, it is only the holes found within the floor of the oval fossa, or in its antero-inferior buttress (Figure 2), which are true defects of the atrial septum.

The larger part of the atrial septum, of course, is provided by the primary septum, or “septum primum”, which forms the floor of the oval fossa. In many of the textbooks currently extant, it remains the fact that the superior rim of the fossa is usually described as the second atrial septum, or the “septum secundum”. Even prior to the turn of the nineteenth century, however, it had been shown that this rim was no more than an infolding of the atrial walls [19]. We now know that the true second atrial septum is formed by muscularisation of the mesenchymal cap carried on the leading edge of the primary septum as it closes the primary atrial foramen, but with this component reinforced by growth of tissues through the dorsal mesocardium in the form of the vestibular spine [20]. It is the failure of fusion of these two components that permits the retention of a channel that, as with the oval fossa, is a true atrial septal defect. This is the vestibular defect, first described by Sharratt and colleagues, Ref. [21] but now described in much greater detail by ourselves, with additional evidence provided to reveal its developmental background [22].

**Figure 3:**
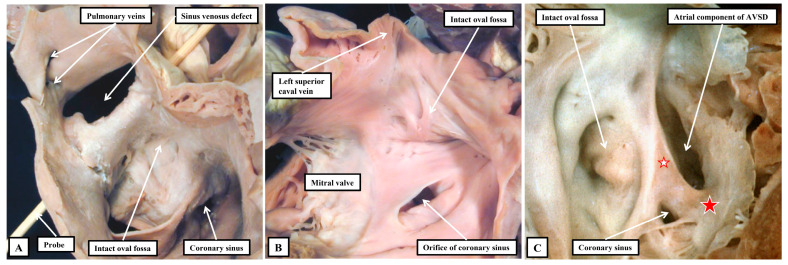
The images show the features of the channels that permit interatrial shunting whilst not being due to deficient atrial septation. (**A**) shows a superior sinus venosus defect, as seen through the opened right atrium. (**B**) shows a coronary sinus defect, shown through the opened left atrium. Note the presence of the connection of a persistent left superior caval vein to the atrial roof. (**C**) shows the so-called “ostium primum” defect, which is an atrioventricular septal defect (AVSD) but with shunting confined at atrial level due to the attachment of the bridging leaflets of the common atrioventricular valve to the crest of the scooped-out ventricular septum. The white star with red borders shows the site of the anticipated atrioventricular node at the apex of the triangle of Koch. This node, however, is unable to give rise to the conduction axis, which arises from an anomalous node formed at the site of union between the scooped-out ventricular septum and the atrioventricular junction (red star with white borders).

It is the channels that permit interatrial shunting whilst not being deficiencies of the atrial septum, however, that are in need of the greatest attention by those seeking to optimise the personalized approach. It follows that, if the lesions themselves are not within the confines of the atrial septum, attention will need to be concentrated on the boundaries of the oval fossa so as to permit accurate recognition. When approached in this fashion, it will become apparent that the so-called “sinus venosus” defect is, in reality, a problem of anomalous connection of the right pulmonary veins (Figure 3A).

In the sinus venosus defect, however, although at least one, and often more, of the pulmonary veins is anomalously connected to one of the caval veins, the pulmonary veins retain their usual connection with the morphologically left atrium. This produces an interatrial veno-venous conduit that provides the channel for the abnormal shunt [23]. Most usually, the anomalous pulmonary veins connect with the superior caval vein, often but not always with the orifice of the caval vein then overriding the intact superior rim of the oval fossa (Figure 3A). Should it be the inferior pulmonary veins that connect anomalously, usually to the inferior caval vein, then the result is to produce an inferior sinus venosus defect [24]. On occasion, however, it can be the middle pulmonary veins that connects anomalously whilst retaining its left atrial connection. This can produce an intermediate variant, which can be difficult to distinguish from a large defect within the oval fossa that extends towards the mouth of the inferior caval vein. It follows that the key to correct diagnosis of all the sinus venosus defects is the finding of anomalously connected pulmonary veins that have retained their left atrial connections.

The rarest type of interatrial communication found outside the confines of the atrial septum is the coronary sinus defect (Figure 3B). It is even rarer to find this variant in isolation. It is a communication through the mouth of the coronary sinus due to the absence of the walls that usually separate the sinus itself from the cavity of the left atrium [25]. Most usually it is associated with persistence of the left superior caval vein, which drains to the roof of the left atrium between the attachments of the left pulmonary veins and the left atrial appendage (Figure 3B). The lesion is part of the spectrum known as “unroofing’ of the coronary sinus [26]. The other more common lesion that permits atrial shunting whilst not involving the atrial septum itself is the so-called “ostium primum” defect [27]. The reason why this defect is not an “atrial septal defect” is more subtle, since it is not that obvious that the lesion itself is not involving the atrial septum (Figure 3C). Careful analysis of the underlying morphology, however, shows that the phenotypic feature is the presence of a common atrioventricular junction. It is the presence of this feature that points to the need to establish the personalized arrangement of the underlying anatomy. This is because, in the setting of the common junction, the left component of the common atrioventricular valve, whilst being a discrete entity guarding the entrance to the left ventricle, is trifoliate, with scant resemblance to the mitral valve. For quite some time, the valve was considered to be no more than a mitral valve with a “cleft” in its anterior leaflet. In reality, the so-called “cleft” is the zone of apposition of the left ventricular components of the leaflets of the common atrioventricular valve that bridge the ventricular septum [28]. Surgical experts recognize these differences, including an understanding that the bifoliate valve created by suturing close this “cleft” remains much different than the bifoliate arrangement seen in the normal mitral valve. The other feature of the so-called “primum” defect is that the bridging leaflets are fused to each other, but additionally fused to the crest of the scooped-out muscular ventricular septum. Indeed, if the leaflets themselves are removed from the heart in the autopsy room, there is no way that the heart can then be distinguished from the more usual variant of atrioventricular septal defect with common atrioventricular junction [29]. The shunting, therefore, is through the atrial component of an atrioventricular septal defect. With careful examination, it can be confirmed that in many examples, the atrial septum is, indeed, intact although oftentimes there are additional deficiencies in the floor of the oval fossa, or on occasion a common atrial chamber, with absence of the atrial septum. In all instances, nonetheless, because of the key presence of the common atrioventricular junction, the atrioventricular node is formed at the site of union with the muscular ventricular septum and the atrioventricular junction. Most usually this is at the crux of the heart, albeit in presence of an analogue of the triangle of Koch in its anticipated location (Figure 3C). The atrioventricular node that gives rise to the conduction axis, therefore, is displaced inferiorly compared to the usual arrangement, which we discuss in greater detail in the next section of our review. As we will show, it is perhaps in the guide that can be obtained to the likely location of the conduction tissues that the personalized approach to diagnosis becomes most important. By the same token, it could well be argued that the differences in morphology make little difference, since the end result is no more than shunting between the atrial chambers. As shown above, however, the recognition of the differences in morphology is crucial in reaching the correct diagnosis. In the sinus venosus defect, for example, it is now recognised that the lesion can only be found when one, or more, of the pulmonary veins is anomalously attached to a systemic vein whilst retaining its left atrial connection. And the recognition that the ostium primum defect is an atrioventricular, rather than an atrial, septal defect provides so much more information over and above the fact that the shunting remains at atrial level, as is the case for the defects within the oval fossa.

### 3.3. The Channels Permitting Ventricular Shunting

Channels permitting interventricular shunting are the commonest congenital cardiac malformations. They can be found in isolation, but they are an integral part of other lesions, such as double inlet ventricles, atrioventricular valvar atresia, tetralogy of Fallot, double outlet right ventricle, and common arterial trunk. They can also be found as associated lesions in the setting of the regular and congenitally corrected variants of transposition, and can be particularly problematic when associated with straddling atrioventricular valves. As was emphasised recently, despite the tremendous advances made in the conduct of congenital cardiac surgery, one of the challenges that remains is the avoidance of iatrogenic heart block [30]. A recent multicenter study reported that approximately 3% of all patients with congenital heart disease undergoing cardiac surgery will develop high-grade atrioventricular block, with 1% requiring permanent pacemaker implantation [31]. And such heart block, should it be inadvertently induced nowadays, is usually the problem encountered during the closure of the channels that permit interventricular shunting. Those pointing to the need to avoid such iatrogenic block are now advocating the use of intraoperative mapping to identify with precision the site of the atrioventricular conduction axis [2,30,32]. Even with the use of their new technique, however, these investigators have yet to eradicate the problem of iatrogenic block [30,32]. This is, perhaps, because as yet their own approach to personalising the diagnostic approach is not as optimal as it might be. As we suggested in our introduction, this may partly reflect that their use of terms to describe the different channels permitting interventricular shunting fail to differentiate the borders of the defect, which is the primary descriptor which relates the defect to the location of the conduction system. Current mapping catheters are limited to spatial resolution between electrodes of approximately 3 to 4 mm. We would argue that the accuracy in predicting the course of the atrioventricular conduction axis in congenital heart malformations which involve ventricular septal defects could further be improved by clinical imaging. Such assessment is now able to provide an in-depth understanding of the components of the so-called central fibrous body, and can reveal how these components are deranged in the setting of various forms of ventricular septal defects, demonstrating how these changes influence the relationship to the conduction system.

**Figure 4:**
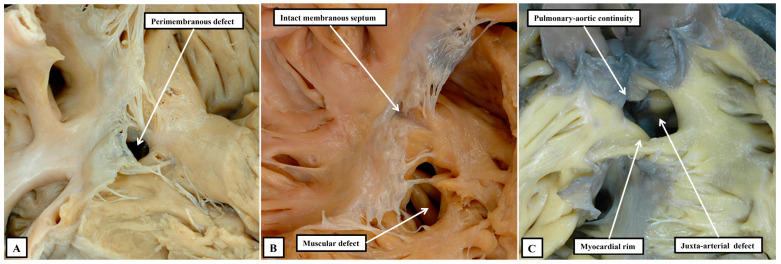
As shown in these images, all channels between the ventricles can be described as being perimembranous (**A**) when there is fibrous continuity between the leaflets of the mitral and tricuspid valves as part of the margin of the defect, muscular (**B**) when the defect has exclusively muscular rims as seen from the right ventricle, or juxta-arterial (**C**) when the defect is roofed by an area of fibrous continuity between the leaflets of the arterial valves, or in the presence of a common arterial trunk by the leaflets of the truncal valve.

We now know that, rather than having three or four parts, Ref. [33] the ventricular septum has only apical muscular and membranous components. During formation of the septum, however, the channel that permits shunting between the developing right and left ventricles undergoes considerable remodelling [34]. When first recognised as a conduit between the developing ventricles, and identifiable as the primary interventricular foramen, all the blood from the developing atrial chambers must pass through the foramen so as to reach the outflow tract, which is supported exclusively above the cavity of the developing right ventricle. With expansion of the atrioventricular canal, the right ventricle achieves its own inlet, with the channel from the left ventricle then providing its outflow tract as the secondary interventricular foramen. With ongoing remodelling, this secondary foramen becomes the definitive subaortic outflow tract, and is never closed. During this process, the embryo builds a shelf in the right ventricle to separate the developing aortic root from the apical part of the cavity. A communication persists, nonetheless, between the space beneath the aortic root and the right ventricular cavity. This is the tertiary interventricular communication, which is closed by tubercles derived from the atrioventricular cushions to complete ventricular septation. This knowledge regarding the process of ventricular septation, when assessed relative to the morphology of the various channels that permit interventricular shunting, Ref. [34] serves to validate the concept that was advanced some time ago. The concept proposed that all such channels could either be perimembranous, muscular, or juxta-arterial (Figure 4) [35].

**Figure 5:**
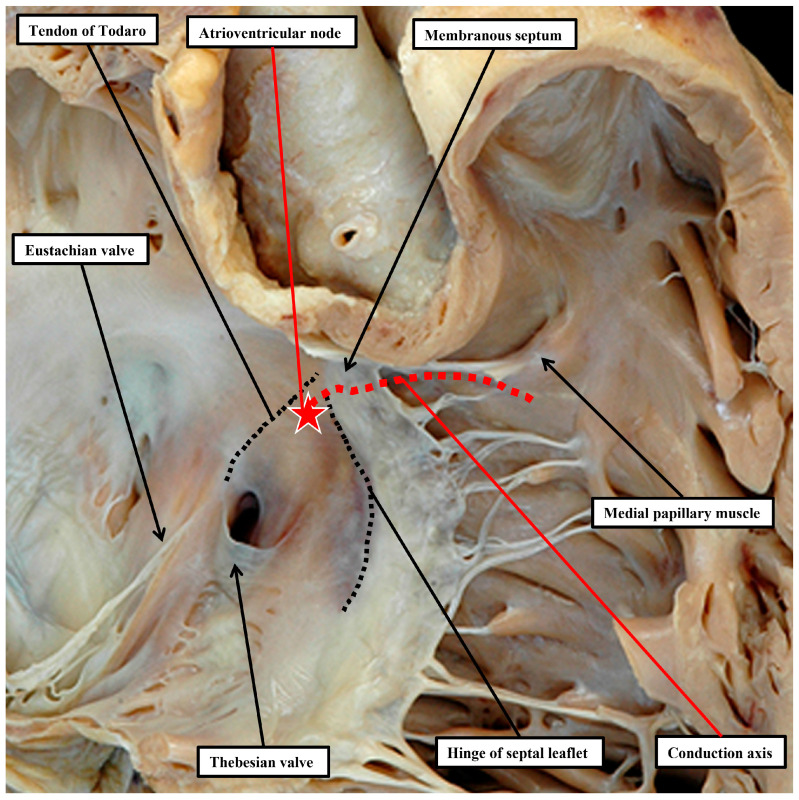
In this heart, showing the right-sided chambers viewed from their right side, the parietal walls of the right atrium and ventricle have been removed to show the septal surfaces. The site of the atrioventricular conduction axis can be predicted with accuracy by drawing a line from the apex of the triangle of Koch, under the inferior margin of the membranous septum, to the site of the medial papillary muscle of the tricuspid valve. The triangle of Koch is delimited by the tendon of Todaro, the continuation intramyocardially of the commissure between the Eustachian and Thebesian valves, and the hinge of the septal leaflet of the tricuspid valve. As shown by the red star with white borders, the atrioventricular node is located in the apex of the triangle. The ventricular component of the conduction axis is then sandwiched between the base of the membranous septum and the crest of the muscular ventricular septum. The right bundle branch emerges as a narrow cord in the environs of the medial papillary muscle. This knowledge, combined with information regarding the phenotypes of ventricular septal defects, permits an accurate estimation of the likely site of the atrioventricular conduction axis (see Figure 6).

We now know that the perimembranous defect represents the unclosed tertiary interventricular foramen [34]. Such defects were initially considered to be “membranous”. The defects themselves, however, are significantly larger than the area occupied by the membranous septum in the hearts with intact ventricular septums. It was Becu and colleagues who pointed out that the defects existed because of deficiencies of septal myocardium in the periphery of the tertiary foramen, hence making them perimembranous rather than membranous [36]. The significance of recognising such defects as being perimembranous is that, with one important exception, the finding indicates that the conduction axis will penetrate through their postero-inferior margin [37]. This information reflects the fact that, in the normal heart, it is possible with accuracy to predict the likely location of the atrioventricular conduction axis as extending from the apex of the triangle of Koch to the inferior margin of the membranous septum, with the right bundle continuing to the site of the medial papillary muscles supporting the zone of apposition between the septal and antero-superior leaflets of the tricuspid valve (Figure 5). A recent study has demonstrated the high reproducibility and accuracy that can now be achieved when using these landmarks to estimate the location of the atrioventricular conduction axis by cardiac computed tomography [38]. Such an approach has already demonstrated a decreased incidence in the occurrence of heart block and need for permanent pacemaker subsequent to surgical intervention in patients with complex congenital left ventricular outflow tract disease [39].

**Figure 6:**
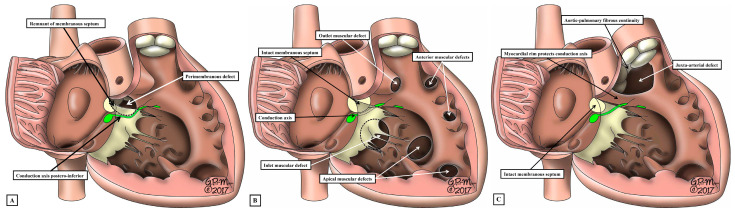
The drawings show how, based on knowledge of the normal arrangement of the atrioventricular conduction axis (see Figure 5), it is possible with accuracy to predict the location of the axis when ventricular septal defects are either perimembranous (**A**), muscular (**B**), or juxta-arterial (**C**). On occasion, however, the juxta-arterial defects can extend to become perimembranous. In the latter eventuality, the conduction axis will be at risk in the postero-inferior margin of the defect, as shown in (**A**).

As part of its course from the apex of the triangle of Koch to the site of emergence of the right bundle branch relative to the medial papillary muscle of the tricuspid valve, the ventricular component of the conduction axis traverses the right wall of the infero-septal recess of the subaortic outflow tract, where it gives rise to the fascicles of the left bundle branch. This means that, when the ventricular septum is deficient due to failure of the membranous septum to close the tertiary interventricular foramen, and providing the atrial septum is normally aligned relative to the inferior component of the muscular ventricular septum, it can be presumed that the axis itself will always penetrate through the area of mitral-to-tricuspid fibrous continuity that forms the postero-inferior border of all perimembranous septal defects (Figure 6A).

**Figure 7:**
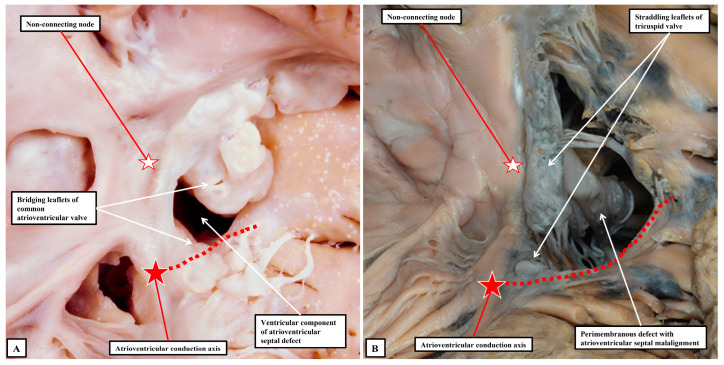
The images show two defects opening to the inlet of the right ventricle with unexpected locations of the atrioventricular conduction axis. (**A**) shows an atrioventricular septal defect with exclusive ventricular shunting, with (**B**) showing the arrangement found when there is straddling of the tricuspid valve.

Although always penetrating postero-inferiorly relative to perimembranous defects, with atrioventricular septal alignment, however, the conduction axis is not always disposed directly on the crest of the muscular ventricular septum. Identification of the defect as being perimembranous, however, does always permit the surgeon to identify the major area of danger during closure of the defect. The knowledge of the normal location of the axis also permits accurate predictions to be made when defects have exclusively muscular rims as seen from the right ventricle (Figure 6B). The key defects in this regard are those that open to the inlet of the right ventricle, since it follows that the conduction axis must extend superiorly relative to such defects. The variability observed when defects are muscular also points to the significance of taking note of their location, in addition to the make-up of their borders [40]. Also of particular importance is the issue of septal malalignment. Most usually, the malalignment involves the arrangement of the muscular or fibrous outlet septum relative to the crest of the muscular ventricular septum. Since the outlet septum never harbours the conduction axis, this is of less importance than malalignment of the apical ventricular septum relative to the leading edge of the atrial septum. In this setting, as when there is a common atrioventricular junction (Figure 3A and Figure 7A), the ventricular part of the conduction axis takes its origin from an atrioventricular node that is no longer found at the apex of the triangle of Koch.

**Figure 8:**
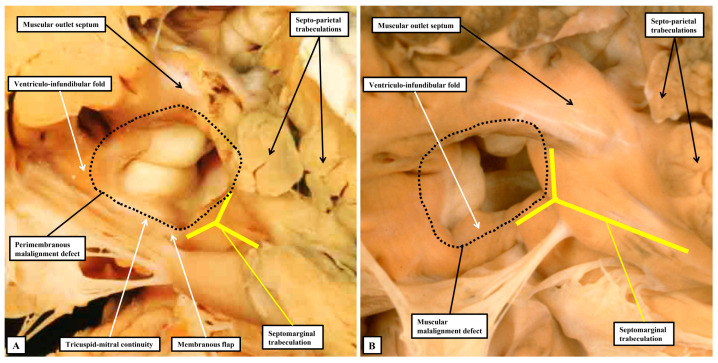
The images show the features of defects as seen in the setting of tetralogy of Fallot, which is characterised by malalignment between the muscular outlet, or conal, septum relative to the crest of the muscular ventricular septum. In (**A**), part of the border of the defect to be closed by the surgeon, shown by the dashed black line, is formed by fibrous continuity between the leaflets of the mitral and tricuspid valves, making the defect perimembranous. As was shown in Figure 6B, the conduction axis in this setting will be at risk in the postero-inferior margin of such a defect. In (**B**), again with malalignment between the outlet septum and the apical muscular septum, the defect to be closed by the surgeon has exclusively myocardial rims due to fusion between the caudal limb of the septomarginal trabeculation and the ventriculo-infundibular fold. The muscle bar thus formed protects the atrioventricular conduction axis.

Even if there is a common atrioventricular junction, as long as there is atrioventricular septal alignment, the conduction axis still reaches to the crux of the heart, with the atrioventricular node formed in the inferior part of the base of the atrial septum. This is not the case when there is atrioventricular septal malalignment (Figure 7B). In this latter setting, there is still fibrous continuity between the leaflets of the mitral and tricuspid valves in the roof of the left ventricle. This means that the septal defect itself can still be defined as being perimembranous. Because of the septal malalignment, however, the axis arises from an anomalous node formed at the point where the inferior part of the muscular ventricular septum meets the atrioventricular junction [41]. Those who advocate the importance of mapping to identify with precision the location of the conduction axis now recognise the significance of septal malalignment. Their system of description of septal defects, however, as far as can be established from their account, does not recognise the particular importance of the perimembranous defect opening to the inlet of the right ventricle when there is atrioventricular septal malalignment. Such defects, in their system, are labelled as being “conoventricular”, or of “atrioventricular canal type”. It is unlikely that the investigators will accurately identify the site of the conduction axis on the basis of pre-existing knowledge when using such inappropriate definitions. Their use of “conoventricular” is also likely to fail to identify the presence or absence of a key structure that determines the location of the conduction axis in the setting of defects opening to the outlet of the right ventricle. In the setting of tetralogy of Fallot, for example, the typical septal defect opens to the outlet of the right ventricle between the limbs of the septomarginal trabeculation, or septal band. It is the divorce between the outlet, or conal, septum and the apical muscular septum seen in these lesions that represents the essence of the “conoventricular” defect [33]. Such defects, however, can be perimembranous (Figure 8A) or can have exclusively muscular rims (Figure 8B).

The difference is highly significant with regard to the disposition, and vulnerability, of the atrioventricular conduction axis. We accept that accurate use of intraoperative mapping will prove to be very helpful in avoiding danger areas during surgical closure of the multiple channels that permit interventricular shunting. With current limitations in spatial resolution of the catheters used, such an approach can only be improved when those undertaking the procedure use a system of classification and description designed to focus on the likely disposition of the atrioventricular conduction axis [34,35]. The importance placed on the need to avoid iatrogenic heart block [30] is itself then an additional reason for placing primacy on categorising defects on the basis of their borders, whilst not ignoring the significance of geography or septal malalignment [42].

### 3.4. The Congenitally Malformed Aortic Root

The personalized approach is achieving ever increasing importance with regard to the treatment of congenital malformations of the aortic root [43,44,45]. This reflects the appreciation, since the turn of the century, that with appropriate diagnosis an increasing number of patients can be treated by repair, as opposed to replacement, of the abnormal root [46]. The success of the procedure depends on an accurate assessment of the components of the malformed root relative to the normal arrangement [47]. This, in turn, demands a full appreciation of the normal anatomy, itself compromised previously by the acknowledged “tower of Babel” existing with regard to uniform description [48]. Despite the best efforts of those involved with the diagnosis of congenitally malformed aortic roots when seen in infants and children, Ref. [49] problems still exist when seeking to make comparisons with the personalized approach as used by those dealing with adult populations [50]. As with all the other lesions we have discussed in our review, our own opinion is that the best way of making progress is to use words for the purposes of description in such a fashion that they will be understood by all, as opposed to those entrenched within their own limited disciplines, who are less inclined to move towards consensus. It can be argued that the emergence of the “tower of Babel”, as identified in the questionnaire conducted by the German surgeons, was the consequence of such entrenchment [51]. How then, can we best describe the aortic root to produce a system that, hopefully, will be acceptable to all, and that will lead to the required personalised approach to diagnosis and treatment?

**Figure 9:**
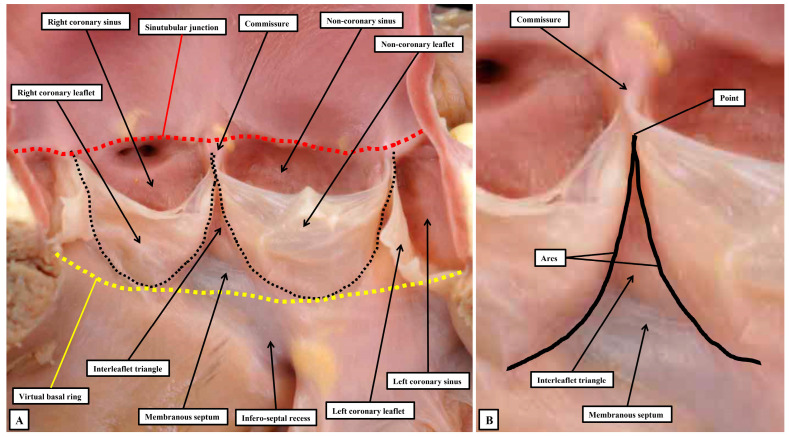
(**A**) shows the aortic root opened through the left coronary sinus and spread to provide the typical view as seen by the morphologist. The boundaries of the root are delimited by the proximal and distal extents of the semilunar lines of attachment of the flaps that, during the cardiac cycle, open and close to guard the exit from the left ventricle. (**B**) is an enlargement of the distal extent of two of the hingelines. The arcs of the hingelines come together at the sinutubular junction to form a point. This point is usually described as the valvar commissure. If named on the basis of the dictionary definition, it would be described as a “cusp” (see text for discussion).

In the first instance, we acknowledge that the view obtained by the morphologist is not necessarily comparable to that provided to those diagnosing clinical problems. When assessing the arrangements of the components of the arterial valvar complexes, the anatomist usually opens the root by making a cut through its circumference, and then spreading the walls so as to reveal its components. Such an approach shows that, in the normal root, there are three relatively thin flaps that move in such a way as to open and close the exit from the left ventricle. During the cardiac cycle, the entirety of the root is in motion. It is the parts that come together so as to close the ventricular exit, nonetheless, that move the greatest, as is also the case with the atrioventricular valves. Of note, the roots guarding the ventricular outlets are themselves frequently described as being “semilunar”, in this way differentiating them from the valves at the ventricular entrances. Examination of the roots in the fashion favoured by the anatomist, however, shows that it is the attachments of the moving components within the root, rather than the overall root itself, which are semilunar. The roots themselves are best distinguished from the atrioventricular complexes by considering them to be arterial. Examination of the arrangement of the semilunar moving flaps then shows that their hingelines come together at the distal extent of the root, which can be recognised as marking the boundary between the tubular intrapericardial arterial component and the walls of the sinuses which support the moving components. The view of the root as opened by the morphologist serves to illustrate some of the ongoing problems relating to the use of words. As emphasised, the major feature of the root related to its function is the hinging in semilunar fashion of the flaps that move to the greatest extent during the cardiac cycle. In the normal root, there are three such moving components, with the hingelines coming together at the distal extent of the root. This distal margin is thickened to form the sinutubular junction [52]. The proximal extent of the root is then marked by a plane which joins together the nadirs of the semilunar hinges. It is the name to be given to this plane that produces one of the ongoing problems in description. Examining the opened root shows that there is no anatomical structure which coincides with its proximal boundary. As indicated, the boundary is a virtual entity. When assessing the opened root, it can be described as the virtual basal ring (Figure 9A), although during life it is usually oval rather than circular.

**Figure 10:**
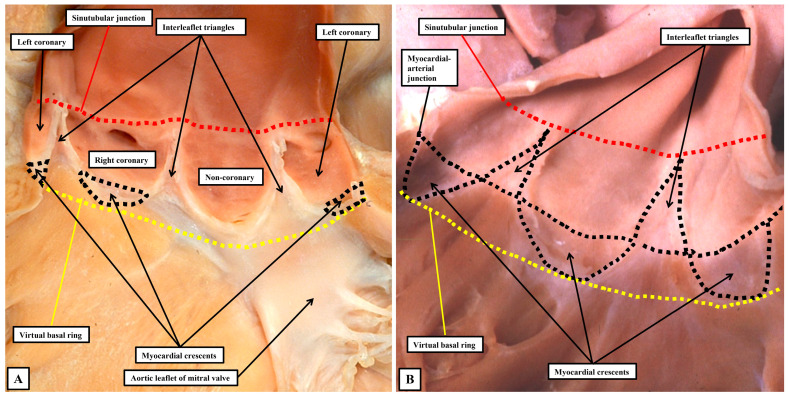
The panels shows the arrangements of the aortic (**A**) and the pulmonary (**B**) roots having removed the larger portions of the components that open and close so as to guard the ventricular exits. It is the distal and proximal extents of the semilunar hingelines of the moving components of the valvar complexes that mark the boundaries of the roots. In the aortic root, which has been opened by making a cut through the left coronary aortic sinus, the nadirs of the hinges of moving parts supported by the sinuses which give rise to the coronary arteries sequester small crescents of myocardium at the bases of both sinuses (**A**). This formation of myocardial crescents is much more obvious in the pulmonary root, where the crescents are formed at the bases of all three valvar sinuses (**B**). This means that, in the pulmonary root, it is possible to recognise a discrete junction between the infundibular myocardium and the arterial walls of the valvar sinuses. In the aortic root, such myocardial-arterial junctions are found only at the bases of the sinuses giving rise to the coronary arteries.

This then creates further problems with nomenclature. It is this plane which is described by echocardiographers as the valvar “annulus”, with many surgeons also using this definition [48]. When assessing the diameter of this “annulus”, echocardiographic measurements are typically shown as extending from the nadir of one semilunar hinge to the nadir of an opposite hingeline. It is obvious that such lines can never show accurately the diameter of the root, irrespective of whether the virtual plane is circular or oval [42]. There is then another problem with this use of “annulus”. Surgeons, when replacing rather than repairing the valve, remove the diseased leaflets. Removal of the diseased leaflets reveals their remaining semilunar remnants. A small majority of surgeons then defines these persisting semilunar remnants as representing the valvar “annulus” [41]. Such usage has the advantage of recognising a true anatomical entity. In terms of hemodynamics, furthermore, it is the semilunar hingelines which mark the lateral junction between the components registering ventricular as opposed to arterial forces during the cardiac cycle [45]. When the valve is closed, ventricular forces can be registered distally to the level of the sinutubular junction, with the measured forces constrained within the ventricular cavity by the walls of the interleaflet triangles (Figure 10).

In terms of the hemodynamics, therefore, the semilunar hingelines of the moving components of the valvar complex create the lateral margins of the ventriculo-arterial junction. One of us had previously described anatomical ventriculo-arterial junctions as existing within the roots, contrasting the location of the perceived anatomical entities with the discrete hemodynamic boundaries [52]. We now recognise that the anatomical junctions would better have been described as being myocardial-arterial. They are much more obvious in the pulmonary as opposed to the aortic root. This is because, in the pulmonary root (Figure 10B), the nadirs of all three semilunar hingelines extend so as to incorporate myocardium at the bases of the valvar sinuses. In the pulmonary root, therefore, it is justifiable to correlate the myocardial-arterial junction with an anatomical ventriculo-arterial junction. This is not the case for the aortic root (Figure 10A). In the aortic root, because of the fibrous continuity between its moving parts and the comparable moving part of the mitral valve, the semilunar hingelines incorporate myocardium only at the bases of the sinuses which give rise to the coronary arteries. It is not possible, therefore, to recognise an obvious anatomical ventriculo-arterial junction in the area of fibrous continuity between the mitral and aortic valve (Compare Figure 10A,B).

**Figure 11:**
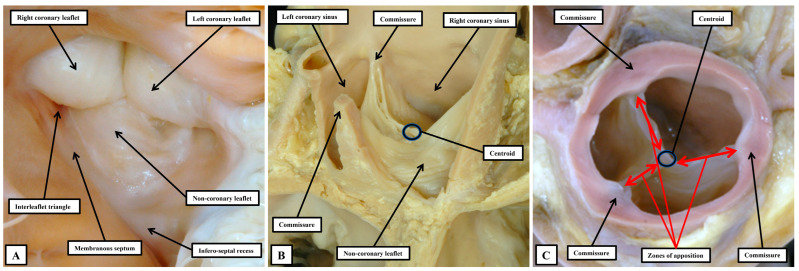
These panels show three views of the intact aortic root, producing images more comparable to those seen by diagnosticians (see Figure 12). In (**A**), the view shows the closed leaflets of the aortic valve as seen from beneath. When seen from this aspect, the ventricular surfaces of the closed leaflets bear a significant resemblance to the cusps of the molar and premolar teeth. In (**B**), dissection to remove the walls of the left coronary and non-coronary valvar sinuses reveals the overall crown-like configuration of the root. (**C**) is prepared by photographing the closed valve from the arterial aspect, having transected the root at the level of the sinutubular junction. It shows the zones of apposition between the closed leaflets, which extend from the so-called valvar “commissures” to the centroid of the closed valvar orifice.

The astute reader will have observed that, thus far when describing the anatomy of the arterial roots, we have assiduously avoided giving a specific name to their major moving components. Our own preference is to describe these major moving parts, along with the major moving components of the atrioventricular valves, as the leaflets. Many, nonetheless, argue that the major moving components of the arterial roots should be described as “cusps”, distinguishing them in this fashion from the atrioventricular valves, which are said to have leaflets. We find such an approach to be illogical both anatomically and linguistically. In the first instance, one of the atrioventricular valves is still universally described as being “tricuspid”. It is highly unlikely that all will accept that the valve should be renamed as being “trileaflet”. In the second place, one of the definitions of a “cusp”, as provided in dictionaries, is the point at which two arcs meet. As we have shown, if such a definition was used logically for description of a part of the arterial roots, it is the commissures which would appropriately be described as the “cusps” (Figure 9B). When the arterial roots are viewed as intact entities, nonetheless, as opposed to being seen when opened, the view of the ventricular aspect of the closed leaflets shows a marked resemblance to the surfaces of the molar and premolar teeth, areas which are conventionally described as “cusps” (Figure 11A).

**Figure 12:**
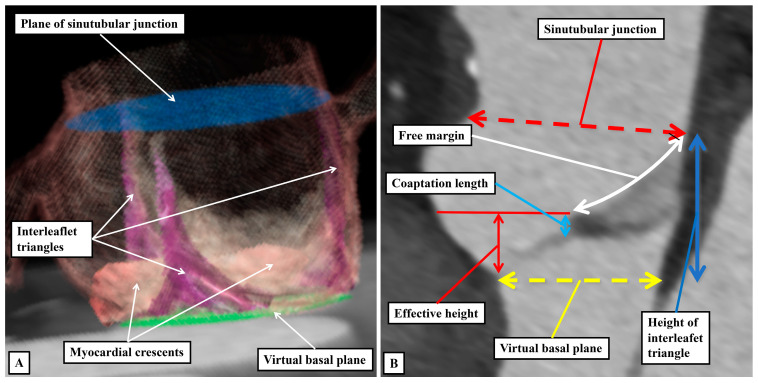
(**A**) shows a three-dimensional reconstruction of the aortic root using a computed tomographic dataset of a normal individual. The interleaflet triangles have been segmented, along with the myocardial crescents at the bases of the valvar sinuses which give rise to the coronary arteries. The planes of the sinutubular junction and the virtual basal ring have also been segmented. (**B**) shows a two-dimensional section of a different computed tomographic dataset taken through the non-coronary sinus. It shows the detailed metrics that can now be taken for the purposes of repair of diseased aortic roots.

It is likely that such similarities prompted the old anatomists to describe the moving components of the valvar complexes as “cusps”. Many, of course, still consider the moving parts to be best described as being “cusps” [51]. Such usage, however, now creates additional problems. In this regard, we accept that malformed aortic roots, in which there are only two, as opposed to three, major moving parts will continue to be described as being “bicuspid”. The purpose of those promoting “cusp” as the best descriptor of the moving parts, however, was to achieve more universal recognition of the different phenotypes that could produce variants with only two major moving components, or in some instances only one major moving part [51]. If such a system is, indeed, to be universally applicable, it must distinguish unequivocally between the major moving parts, and the components of the aortic root that support them. It is unfortunate, therefore, that electrophysiologists now describe how their efforts to cure arrhythmias arising from the ventricular outflow tracts involves ablation of the valvar cusps [53,54]. It is highly unlikely that such practitioners would be seeking to ablate the moving components. It is the myocardial crescents found at the bases of the valvar sinuses that are the substrates for such arrhythmias. Even those promoting the restriction of the use of “cusp” to the moving components incorrectly described the coronary arteries as arising from the “cusps”, Ref. [51] when it is obvious that they arise from the sinuses. The key to correct personalized description, therefore, is to distinguish between the major moving components of the valves and the walls of the parts of the root that support them. This can no longer adequately be achieved when both components are described by different sets of practitioners as being the “cusps”. The solution is to avoid the use of “cusp”. A distinction can then be made between the leaflets and their supporting sinuses. A case can be made, on linguistic grounds, for naming the valvar commissures as the cusps. This would produce still further confusion. The term “commissure” is now entrenched as representing the peripheral attachments of the leaflets at the sinutubular junction. For anatomists, nonetheless, commissures are the junctions between adjacent entities, as in the eyes or the lips, or between the bones of the skull. When considering the arterial valvar complexes, attention does need to be directed to the areas over which the leaflets coapt, which ideally would be named as “commissures”. To avoid further confusion, we describe these areas as the zones of apposition between the leaflets (Figure 11C). We also take particular note of the walls of the interleaflet triangles, which extend the ventricular cavity to the sinutubular junction when the leaflets are closed. Such distinction between leaflets, sinuses, and interleaflet triangles is now easy to make when the aortic root is displayed using three-dimensional reconstructions of computed tomographic images. By segmentation of the three-dimensional datasets, it is also possible to show the extent of myocardium incorporated at the bases of the valvar sinuses which give rise to the coronary arteries (Figure 12A). By using two-dimensional sections from such computed tomographic datasets, it is possible to measure the dimensions that should ideally be replicated during surgical repair of diseased roots (Figure 12B).

**Figure 13:**
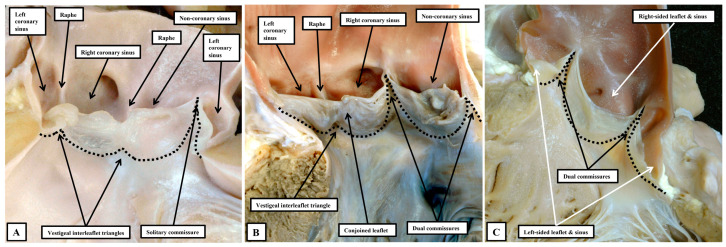
The panels show how the malformed aortic root is best described according to the number of its sinuses, with the skirt of persisting leaflet tissue effectively producing a bileaflet or unileaflet arrangement according to the number of zones of apposition within the skirt that extend to reach the sinutubular junction. (**A**) shows the so-called “unicuspid and unicommissural arrangement”, which is built on a trisinuate scaffold. (**B**) shows the classical bicuspid variant, with fusion of the leaflets supported by the two valvar sinuses giving rise to the coronary arteries. This variant is also trisinuate. (**C**) shows the rare variant in which each leaflet is supported by its own sinus, with no evidence of fusion of developmental primordiums. This variant is bileaflet and bisinuate.

It is then the distinction between the number of sinuses, leaflets, and in particular the interleaflet triangles, which serves to demonstrate the phenotypes making up the so-called “bicuspid aortic valve” [49]. It has long been recognised that this lesion, in most instances, is the consequence of fusion during the fetal period of two of the developing leaflet primordiums [55]. In most instances, it is the leaflets that are supported by the sinuses giving rise to the coronary arteries that fuse during development. It is also frequent to find examples in which the leaflet guarding the developing right coronary sinus has fused with the leaflet destined to guard the non-coronary sinus. Fusion of the developing primordiums of the non-coronary and left coronary sinuses, in contrast, is exceedingly rare [56]. In a well-recognised categorisation, however, the variant in which the moving components had effectively fused into a solitary skirt of leaflet tissue had also been included as a variant of the “bicuspid” arrangement [57]. Analysis of the phenotypes on the basis of the numbers of sinuses, leaflets, and interleaflet triangles points to the deficiency of the latter approach. In the so-called “unicuspid and unicommissural variant”, examination from the ventricular aspect reveals the presence of two vestigial interleaflet triangles, which correspond with raphes in the skirt of persisting leaflet tissue seen when the arterial aspect is interrogated. There is but a solitary sone of apposition within the skirt that extends to the level of the sinutubular junction. Analysis on the basis of the number of raphes and interleaflet triangles thus shows that the variant is unileaflet, but trisinuate. The three well-recognised variants of the classical bicuspid valve can then all be seen to be bileaflet, but built again on a trisinuate scaffold. Interrogation in this way then reveals the presence of yet a further series of phenotypes (Figure 13).

In these rare variants, there are two leaflets, but with each leaflet supported by its own sinus in the absence of vestigial interleaflet triangles or raphes [58]. In these bisinuate and bileaflet variants, the leaflets themselves can either be positioned in antero-posterior fashion, with both coronary arteries arising from the same sinus, or in right-left fashion, with a coronary artery arising from each sinus. The inference can be made that the variants reflect failure of formation, during development, of one of the swellings that remodel to give rise to the leaflets and their supporting sinuses [59]. It is the measurement of the dimensions of the sinuses and leaflets, and the extent of the interleaflet triangles, which provides the means of customising surgical repair [47]. This, in turn, is the essence of the personalized approach, which in turn brings all the discussions of anatomy into clinical relevance.

## 4. Discussion

In our review, we have sought to emphasize how a personalized approach is now feasible during the diagnosis of congenital cardiac malformations. There are, of course, multiple lesions that result from disordered cardiac development prior to birth. In the recent 11th iteration of the International Classification of Disease, over 300 definitions have been provided for the various phenotypes [1]. From this extensive list, we have chosen but four groups of lesions to illustrate the validity of using a personalized approach for their diagnosis. These lesions are, first, those which underscore the recognition of the different basic phenotypes that can exist at the level of the atrial chambers. Such recognition of atrial arrangement is the starting point for sequential segmental analysis [4]. As we have shown, it also conveys crucial information regarding the location of the conduction tissues. We have then shown that neither interatrial nor interventricular communications are as straightforward as might be imagined, in that the variability in phenotypes has been recognized for centuries. It is simple attention to the underlying anatomy, and knowledge of the extent of the true septal components, that underscores the personalized approach. The same goes for the variability now known to exist within the so-called “bicuspid” valve. In this latter group of lesions, the use of words in their everyday fashion is of particular importance in providing the universal understanding that is essential if we are properly to adopt the personalized approach. Change does not happen overnight. In the fullness of time, simplicity will prove to be a major determining feature. And simplicity requires that words be used in such a way that they are understood by all, as opposed to those making up the small cohorts who seek to retain their own individuality.

The importance of accurately describing congenitally malformed hearts is highlighted in numerous recent publications. The role of machine learning to inform the diagnosis depends on automated classification systems for congenital heart disease [60]. Advanced clinical prediction models fundamentally rely on precise anatomical terms in order to prevent their instability in the multiverse and the quality of the model [61]. Moreover, computational technologies such as artificial intelligence and extended reality require mining data collections of invasive and non-invasive imaging modalities such as echocardiography, computer tomography, angiography, and magnetic resonance. Reconstructing anatomies and facilitating preprocedural planning cannot be obtained unless there is a clear description of the cardiac anatomy [62]. Producing a unified anatomical description in normal and malformed hearts has facilitated better outcomes in structural interventions in acquired heart disease. This has been demonstrated in coronary interventions, transcatheter aortic and pulmonary valve replacement, left atrial appendage occlusion, ablative procedures for arrhythmias to name just a few [63,64,65,66,67]. The use of 3D segmentation, analysis and printing for congenital heart disease with the aim to facilitate education, research, training and surgical simulation is only being possible with an accurate description of anatomy because advanced imaging reveals more and more anatomical details [68,69,70,71,72,73].

## Data Availability

No new data was created specifically for the purpose of our review.
